# Using Genotyping and Geospatial Scanning to Estimate Recent *Mycobacterium tuberculosis* Transmission, United States

**DOI:** 10.3201/eid1803.111107

**Published:** 2012-03

**Authors:** Patrick K. Moonan, Smita Ghosh, John E. Oeltmann, J. Steven Kammerer, Lauren S. Cowan, Thomas R. Navin

**Affiliations:** US Centers for Disease Control and Prevention, Atlanta, Georgia, USA

**Keywords:** tuberculosis and other mycobacteria, transmission, genotyping, geospatial, clustering, molecular epidemiology, Mycobacterium tuberculosis, bacteria, respiratory infections

## Abstract

To determine the proportion of reported tuberculosis (TB) cases due to recent transmission in the United States, we conducted a cross-sectional study to examine culture-positive TB cases with complete genotype results (spoligotyping and 12-locus mycobacterial interspersed repetitive unit–variable-number tandem repeat typing) reported during January 2005–December 2009. Recently transmitted cases were defined as cases with matching results reported within statistically significant geospatial zones (identified by a spatial span statistic within a sliding 3-year window). Approximately 1 in 4 TB cases reported in the United States may be attributed to recent transmission. Groups at greatest risk for recent transmission appear to be men, persons born in the United States, members of a minority race or ethnic group, persons who abuse substances, and the homeless. Understanding transmission dynamics and establishing strategies for rapidly detecting recent transmission among these populations are essential for TB elimination in the United States.

## Abstract 2

These tools may enable direction of resources to populations with high transmission rates.

Molecular characterization of *Mycobacterium tuberculosis* complex has been available for >2 decades in the United States. As a tool to enhance programmatic activities, tuberculosis (TB) genotyping is a useful adjunct to epidemiologic field investigations by defining outbreaks (*1**,**2*), discerning episodes of reactivation and relapse (*3**,**4*), confirming suspected laboratory contamination (*5**,**6*), and evaluating and monitoring TB control program performance (*7*). TB genotyping results, when combined with epidemiologic data, help identify persons with TB disease who are involved in the same chain of recent transmission (*8*). Previous analytic studies have used TB genotyping data in conjunction with epidemiologic data to assess correlates of recent TB transmission within localized populations (*9**–**15*). A basic assumption of this approach is that recent TB transmission is localized in place and time, that is, progression to TB disease from an infection acquired within the past few years and in the same jurisdiction.

Population-based molecular epidemiologic studies are often subject to several biases and methodologic limitations that impede the ability of investigators to make valid statements about recent TB transmission events in the absence of direct data regarding interpersonal contacts (*16*). Estimating recent TB transmission is often limited by abbreviated study periods, convenience isolate sampling, and ambiguous geographic boundaries defined for jurisdictional or geopolitical reasons (*17**,**18*). TB transmission is not likely to be bound by these artifacts, however. Spatial scanning to detect disease clusters has been successfully applied in multiple settings and for various diseases (*19*). Using this method in a multiyear, nationally representative database of both genotype and routinely collected TB surveillance data may offer a better solution for accurately defining recent TB transmission.

In 2004, the US Centers for Disease Control and Prevention (CDC) offered universal access to TB genotyping through the National Tuberculosis Genotyping Service (NTGS) to routinely characterize at least 1 *M. tuberculosis* complex isolate from every TB case-patient in the United States (*20*). Although the intent of this system is to support local TB programs for public health action, data collected from this system offer a unique opportunity to explore and describe the molecular epidemiology of TB and establish comprehensive molecular TB surveillance in the United States. In this analysis, our goals were to estimate the proportion of TB in the United States attributable to recent transmission and to assess clinical, demographic, and epidemiologic factors associated with recent TB transmission.

## Methods

### Study Population

This study includes verified cases of TB reported to the US National Tuberculosis Surveillance System (NTSS) by the 50 states and the District of Columbia. Clinical, demographic, and epidemiologic variables for each case-patient are collected for surveillance purposes and are described elsewhere (*21*). *M. tuberculosis* complex isolates were characterized by using a standardized protocol for spacer oligonucleotide typing (spoligotyping) and 12-locus mycobacterial interspersed repetitive unit–variable-number tandem repeats (MIRU-VNTRs) (*22*). NTGS results for each submitted isolate were linked to NTSS case records by state and local TB control programs; a standardized case identification number and a unique laboratory accession number were used to form discrete individual isolate-case records (*20*). When multiple isolates were genotyped for the same person in the same surveillance year, case-patients with discordant genotyping results were excluded from analysis for clustering assignment and risk factor analysis. The final study population included all persons with verified culture-positive TB cases reported during January 2005–December 2009 with a complete spoligotype and 12-locus MIRU-VNTR result.

Four major phylogenetic lineages for *M. tuberculosis,* along with speciation of *M. africanum* and *M. bovis,* were identified by using spoligotyping motifs that referred to an international standard (*23*). Substance abuse was defined by using previously published methods (*24*). Persons with TB who received a positive HIV test result at the time of TB diagnosis were classified as TB/HIV case-patients. Persons with TB and negative HIV results or unknown HIV status were classified as having non-HIV TB.

### Genotype and Geospatial Clustering

Genotype clusters were defined as cases with matching spoligotype and 12-locus MIRU-VNTR results (i.e., exact match on all loci) reported within statistically significant geospatial zones determined by a spatial scan statistic (*25*). SaTScan version 9.1.0 (*26*) was employed to identify geographic areas with a larger-than-expected rate of discrete genotype clustering, and all other culture-positive TB cases counted during the study were considered as the background rate. In brief, all cases were aggregated by genotype according to residential ZIP code where they were reported. Each genotype was then scanned separately, applying a purely spatial analysis, in which the number of events in an area was assumed to be Poisson-distributed to generate circular zones of various sizes up to a maximum radius of 50 km. An evaluation of outbreak investigations conducted by CDC demonstrated no difference in cluster membership when 50-km and 100-km SaTScan search radii were used to identify known epidemiologically linked genotype cases (CDC, unpub. data).

A log-likelihood ratio was calculated for each zone in comparison with all possible zones, with the maximum likelihood ratio representing the zone most likely to identify spatial clustering for each genotype. A Monte Carlo simulation with 999 repetitions was used to determine the distribution of the scan statistic under the null hypothesis of spatial randomness; significant spatial clusters were chosen at an α of p<0.05. Three scans comprised of 3-year overlapping intervals (scan A, 2005–2007; scan B, 2006–2008; scan C, 2007–2009) were performed to identify spatial clusters occurring within a 3-year period. If cases were identified as a member of a statistically significant spatial cluster in any of the 3 periods, they were considered clustered. No duplicative case counting occurred. The purpose of this spatial scan was to characterize each case for a dichotomous outcome: clustered or not clustered. Cases that were both genotypically and spatially clustered were considered recent TB transmission for the purposes of this study. All cases that were not genotypically and spatially clustered were considered reactivation of remotely acquired TB infection, or reactivation TB. For comparative purposes, national-, state- and county-level clustering definitions were created. National-level clustering was defined as >2 culture-positive cases with identical genotypes reported anywhere in the United States during 2005–2009. State-level clustering was defined as >2 culture-positive cases with identical genotypes reported from the same state during 2005–2009. County-level clustering was defined as >2 culture-positive cases with identical genotypes reported from same county during 2005–2009.

### Statistical Analyses

A predictive logistic regression model was used to determine potential associations between clinical (e.g., sputum-smear status, known HIV positivity, site of disease and cavitation on chest radiograph, and previous TB diagnosis) and demographic and risk characteristic variables (e.g., race/ethnicity, age, country of birth, homelessness, substance abuse, incarceration at time of diagnosis, and residence at long-term care facility at diagnosis) and the outcome of interest: geospatial and genotype clustering as a proxy for recent TB transmission. Univariate analysis of the categorical independent variables was done by using Pearson χ^2^. Any variable with a significance value of <0.20 was included in a best subset, multivariate logistic regression model. We built our final model using backward elimination of nonsignificant independent variables (p>0.01). The log-likelihood ratio was used to assess the overall significance of the final models, and the Hosmer-Lemeshow statistic was used to evaluate the fit of each of the final models. To test the hypothesis that factors associated with recent TB transmission events varied by geographic region of the United States, an additional 4 independent models were created following the same process but subset to western, midwestern, northeastern, and southern states, respectively (*27*).

## Results

### TB Case Population

**Figure 1:**
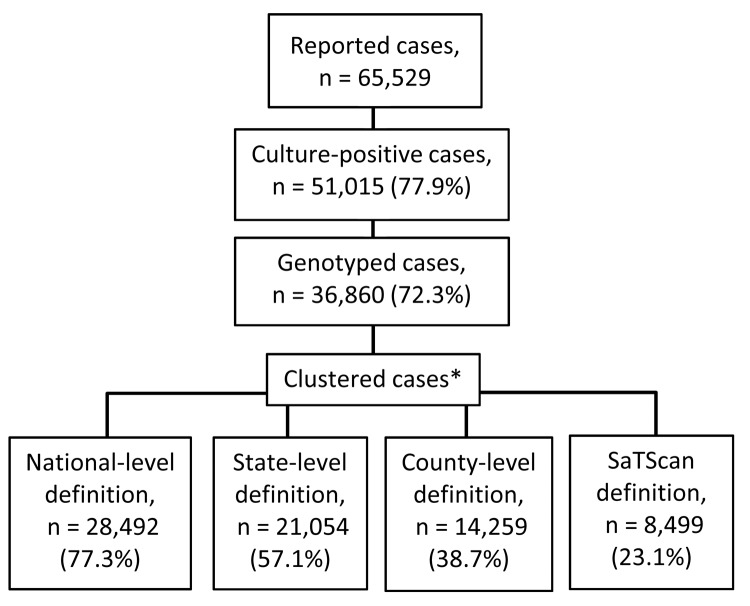
Number of reported cases of tuberculosis, including culture-positive cases, genotyped cases, and genotype clusters, USA, 2005–2009. *Indicates >2 cases with *Mycobacterium tuberculosis* isolates with identical spoligotype and 12-locus mycobacterial interspersed repetitive unit–variable-number tandem repeat analysis results.

During 2005–2009, a total 65,529 verified cases of TB were reported to CDC. Of these, 51,015 (77.9%) were culture-positive (Figure 1). During this period, the overall incidence of TB in the United States declined from 4.8 to 3.8 per 100,000 persons, representing a decline of 20.1% in the overall case count (*21*).

### TB Isolates and Genotype Clusters

During 2005–2009, a total of 45,188 isolates were submitted to NTGS for molecular characterization; 39,474 (87.4%) were successfully matched to a case-patient with reported TB. Two hundred seventy isolates (0.7%) had incomplete results on spoligotype, MIRU-VNTR, or both; 344 case-patients (0.9%) had multiple isolates with discordant genotyping results and were excluded from the analysis. The total number of genotyped TB cases available for analysis was 36,860, representing 72.3% of all reported culture-positive cases. The proportion of reported case-patients for whom complete genotype results were available increased over time, with 6,863 (62.7%) of 10,953 in 2005 and 7,845 (88.4%) of 8,876 in 2009. The number of individual genotype strains (i.e., distinct spoligotype and 12-locus MIRU-VNTR combinations) identified over the study period was 11,722. The proportion of new strains identified per year gradually decreased over time. In 2006, 40.7% of strains identified were new; this percentage was reduced to 14.2% in 2009 (data not shown).

**Figure 2:**
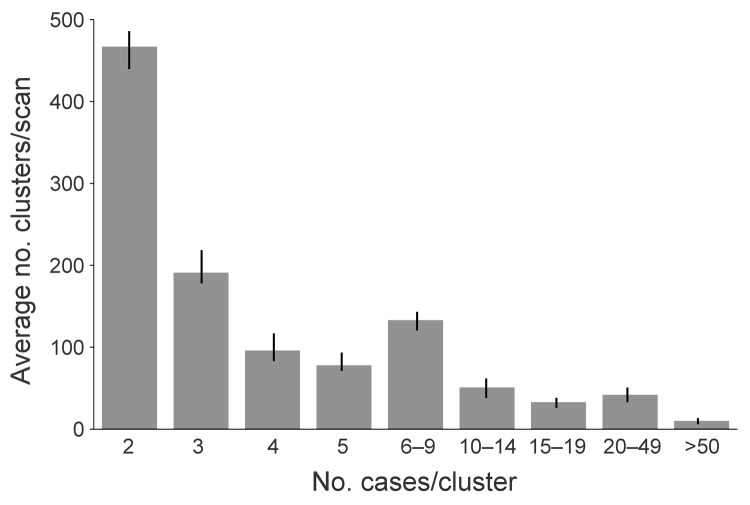
Frequency of genotype clusters of tuberculosis, by cluster size (mean 5.68, median 3, range: 2–173), United States, 2005–2009. Frequency was determined by using SaTScan version 9.1.0 (*26*) on the basis of 3 consecutive, overlapping years: scan A, 2005–2007 (n = 970); scan B, 2006–2008 (n = 1,019); scan C, 2007–2009 (n = 1,128). Error bars indicate upper and lower limits of clusters identified between scan periods.

Of the 36,860 cases for which genotyping had been performed, 8,499 (23.1%) were considered clustered by both genotype and spatial concentration and therefore were thought to be members of a putative recent TB transmission event. The average number of spatially concentrated genotype clusters identified per 3-year scanning period was 1,039 (range 970–1,128). Nationally, the overall mean cluster size was 5.7 members (range 2–173 members) (Figure 2). The median cluster size was 3 members, and almost half (46.1%) of the clusters had only 2 members. Other clustering definitions that use geopolitical boundaries had higher average clustering percentages when the same 3-year window periods were used (national-level, 77.3%; state-level, 57.1%; county-level, 38.7%) (Figure 1).

### Characteristics of Members with Putative Recent TB Transmission

Cluster members were more likely to be male (66.3% vs. 61.7%), to have been born in the United States (57.4% versus 34.4%), to abuse substances (28.4% versus 14.8%), and to have a history of homelessness (11.1% versus 5.0%) than those thought to have reactivation TB (Table 1). The proportion of cluster members also varied by race compared with those with cases due to reactivation TB: Asian, non-Hispanic (17.6% vs. 29.8%); black, non-Hispanic (39.7% versus 21.6%); Hispanic (25.5% versus 28.5%); and white, non-Hispanic (15.0% versus 17.9%).

**Table 1:** Demographic, behavioral, and clinical characteristics of persons involved in putative recent TB transmission events, United States, 2005–2009*

Variable	No. (%) clustered†	Total no. available	Crude OR (99% CI)	Adjusted OR (99% CI)‡
Sex, n = 36,852				
M	5,634 (66.3)	23,136	**1.2 (1.1–1.3)**	1.1 (1.0–1.2)
F	2,862 (33.7)	13,716		
Age group, y, n = 36,860				
0–4	206 (02.4)	395	**3.5 (2.7–4.6)**	**3.1 (1.4–6.8)**
5–14	99 (01.2)	333	**1.4 (1.0–1.9)**	1.2 (0.8–1.9)
15–24	1,131 (13.3)	4,488	1.1 (1.0–1.2)	1.1 (1.0–1.3)
25–44	2,975 (35.0)	12,688	Referent	
45–64	3,025 (35.6)	11,247	**1.0 (1.1–1.3)**	1.0 (0.9–1.0)
>65	1,063 (12.5)	7,709	**0.5 (0.5–0.6)**	**0.5 (0.4–0.6)**
Race/ethnicity, n = 36,761				
American Indian/Alaska Native	111 (01.3)	498	**1.4 (0.9–1.5)**	0.8 (0.6–1.2)
Asian, non-Hispanic	1,496 (17.6)	9,922	**0.7 (0.6–0.8)**	**1.5 (1.3–1.8)**
Black, non-Hispanic	3,368 (39.7)	9,471	**2.2 (2.0–2.4)**	**2.4 (2.2–2.7)**
Hispanic/Latino	2,165 (25.5)	10,238	1.1 (1.0–1.2)	**1.7 (1.5–2.0)**
Native Hawaiian/Pacific Islander	59 (0.7)	190	**1.8 (1.2–2.7)**	**2.6 (1.5–4.4)**
White, non-Hispanic	1,272 (15.0)	6,335	Referent	§
Other	16 (0.2)	107	0.7 (0.3–1.4)	0.9 (0.4–2.2)
Country of birth, n = 36,745				
United States	4,871 (57.4)	14,594	**2.6 (2.4–2.7)**	**2.4 (2.1–2.7)**
Non–US-born	3,611 (42.6)	22,151		
In US <2 y	1,100 (30.5)	7,675	Referent	**
In US 2–5 y	531 (14.7)	3,326	1.1 (1.0–1.3)	
In US >5 y	1,980 (54.8)	11,150	**1.3 (1.2–1.4)**	
Homelessness within past 12 mo, n = 36,558				
Yes	946 (11.2)	2,361	**2.4 (2.1–2.7)**	**1.4 (1.2–1.6)**
No	7,488 (88.8)	34,197		
Substance abuse within past 12 mo, n = 36,860				
Yes	2,415 (28.4)	6,623	**2.3 (2.1–2.5)**	**1.4 (1.3–1.7)**
No	6,082 (71.6)	30,237		
Residence at correctional facility at time of diagnosis, n = 36,815				
Yes	369 (04.3)	1,400	1.2 (1.0–1.4)	0.8 (0.7–1.0)
No	8,121 (95.7)	35,415		
Residence at a long-term care facility at time of diagnosis, n = 36,801				
Yes	180 (02.1)	800	1.0 (0.8–1.2)	NS
No	8,304 (97.9)	36,001		
Reported HIV status, n = 36,860				
Positive	741 (08.7)	2,290	**1.7 (1.5–1.9)**	1.1 (1.0–1.3)
Not positive	7,756 (91.3)	34,570		
Clinical presentation of TB, n = 36,779				
Pulmonary only	6,653 (78.4)	27,083	**1.6 (1.5–1.8)**	1.2 (1.0–1.4)
Extrapulmonary only	992 (11.7)	5,973	Referent	
Pulmonary and extrapulmonary	841 (09.9)	3,723	**1.5 (1.3–1.7)**	1.0 (0.8–1.3)
Sputum smear positivity, n = 31,625				
Yes	4,640 (61.5)	17,934	**1.3 (1.2, 1.4)**	1.1 (1.0–1.2)
No	2,903 (38.5)	13,691		
Cavitary chest radiograph, n = 31,382				
Yes	2,782 (36.8)	10,411	**1.2 (1.2–1.3)**	1.0 (0.9–1.1)
No	4,771 (63.2)	20,971		
Previous TB diagnosis, n = 36,544				
Yes	426 (05.0)	1,680	1.1 (1.0–1.3)	NS
No	8,010 (95.0)	34,864		
*Mycobacterium tuberculosis* or other species spoligotype-based lineage, n = 36,458				
East African Indian	197 (02.3)	1,582	Referent	
East Asian	1,259 (14.9)	4,924	**2.4 (1.9–3.0)**	**1.9 (1.5–2.6)**
Euro-American	5,857 (69.2)	23,441	**2.3 (1.9–2.8)**	**1.5 (1.2–2.0)**
Indo-Oceanic	1,000 (11.8)	5,760	**1.5 (1.2–1.8)**	**1.7 (1.3–2.3)**
* M. africanum*	25 (0.3)	179	1.1 (0.6–2.0)	0.8 (0.4–1.7)
* M. bovis*	127 (01.5)	572	**2.0 (1.4–2.8)**	**2.0 (1.3–3.2)**

*n values indicate number of persons for whom information in category was available. TB, tuberculosis; OR, odds ratio; NS, not significant. **Boldface** indicates significance at α = 0.01. †Genotype clustering: >2 cases with identical spoligotype and 12-loci mycobacterial interspersed repetitive unit–variable-number tandem repeat genotypes occurring within a geospatially concentrated area identified by SaTScan (*20*). Cases must have clustered at least once in 1 of 3 consecutive, overlapping years (scan A, 2005–2007; scan B, 2006–2008; scan C, 2007–2009) (scan windows spatially limited to 50 km). ‡Final model, n = 31,382. §Not included in the model to include both US-born and non–US-born case-patients.

Cluster members with recent TB transmission events were also more likely to have reported HIV-positive results (8.7% versus 5.5%), pulmonary disease exclusively (78.4% versus 72.2%), and positive sputum smear results (61.5% versus 55.3%) and to have had a cavitary chest radiograph at time of diagnosis (36.8% versus 32.2%) than those thought to have reactivation TB. Of the 8,499 persons with cases believed to be caused by recent TB transmission, only 2.1% and 4.4% resided in a long-term care or correctional facility at the time of diagnosis, respectively.

### Genotype Lineage and Recent TB Transmission Events

The proportions of isolates in each phylogenetic lineage were as follows: Euro-American, 64.2%; Indo-Oceanic, 15.4%; East-Asian, 13.5%; East-African/Indian, 4.3%. *M. bovis* isolates accounted for 1.6% of reported cases of TB. Seventy-two percent of reported case-patients with *M. bovis* isolates were non–US-born. *M. africanum* isolates were identified among 179 patients (0.5%), with 88.6% non–US-born. Among members with recent TB transmission events, 69.2% had TB isolates with Euro-American lineage; 14.9% had isolates with East-Asian lineage, 11.8% had isolates with Indo-Oceanic lineage, 2.3% had isolates of East-African/Indian lineage, 1.5% had *M. bovis* isolates, and 0.3% had *M. africanum* isolates.

### Factors Associated with Putative Recent TB Transmission Events

In our final adjusted model, the following odds ratios were noted for variables significantly associated with a higher odds of having a case attributed to putative recent TB transmission (Table 1): age (0–4 years of age: adjusted odds ratio [aOR] 3.1, 99% CI 1.4–6.8); black, non-Hispanic (aOR 2.4, 99% CI 2.2–2.7); Hispanic (aOR 1.7, 99% CI 1.5–2.0); Native Hawaiian/Pacific Islander (aOR 2.6, 99% CI 1.5–4.4); US-born (aOR 2.4, 99% CI 2.1–2.7); homeless persons (aOR 1.4, 99% CI 1.2–1.6); persons who abuse substances (aOR 1.4, 99% CI 1.3–1.7); East-Asian lineage (aOR 1.9, 99% CI 1.5–2.6); and Indo-Oceanic lineage (aOR 1.7, 99% CI 1.3–2.3).

### Geographic Variation Associated with Recent TB Transmission

Best-fit models to predict those with recent TB transmission were conducted for each of the 4 US geographic regions. Many of the main effects associated with recent TB transmission remained constant (US-born, substance abuse, homeless), although factors varied in both magnitude and risk factor across the United States (Table 2).

**Table 2:** Demographic, behavioral, and clinical characteristics of persons involved in putative recent TB transmission events, by location, United States, 2005–2009*

US Census region, no. (%) persons,† and main characteristics	Odds ratio (99% CI)	Wald p value
West, 11,550 (31.3)		
*Mycobacterium bovis* TB	4.4 (2.2–8.8)	<0.0001
US-born	2.4 (4.3–4.7)	<0.0001
East-Asian phylogenetic lineage TB	2.4 (1.4–4.2)	<0.0001
Hispanic	2.3 (1.7–3.0)	<0.0001
Homeless‡	1.9 (1.5–2.9)	<0.0001
Midwest, 10,502 (28.5)		
US-born	2.5 (2.1–3.1)	<0.0001
Black, non-Hispanic	2.1 (1.7–2.6)	<0.0001
Substance abuser§	1.4 (1.3–1.6)	<0.0001
Northeast, 6,090 (16.5)		
Hispanic	2.1 (1.5–3.0)	<0.0001
East-Asian phylogenetic lineage TB	2.0 (1.1–3.6)	0.001
US-born	1.6 (1.2–2.1)	<0.0001
Substance abuser§	1.6 (1.2–2.1)	<0.0001
South 8,718 (23.7)		
Black, non-Hispanic	3.6 (1.5–8.6)	<0.0001
US-born	3.2 (2.5–4.2)	<0.0001
Euro-American phylogenetic lineage TB	2.2 (1.1, 4.3)	0.004
Substance abuser§	1.5 (1.1–4.3)	<0.0001
Total, 36,860		

*TB, tuberculosis. †Column percentage. ‡Self-reported homelessness within 12 mo preceding TB diagnosis. §Self-reported excess alcohol consumption, injection drug use, or noninjection drug use within 12 mo preceding TB diagnosis.

Ethnic disparities for recent TB transmission were found among black, non-Hispanic persons living in midwestern and southern states (aOR 2.1, 99% CI 1.7–2.6; aOR 3.6, 99% CI 1.5–8.6), whereas Hispanic persons had the highest odds among those living in northeastern (aOR 2.3, 99% CI 1.7–8.8) and western states (aOR 2.1, 99% CI 1.5–3.0).

Phylogenetic lineage also varied among the different regions. Euro-American lineage (aOR 2.2, 99%,CI 1.1–4.3) had the strongest association for recent transmission in the south, whereas the East-Asian lineage was most strongly associated with recent transmission in western (aOR 2.4, 99% CI 1.4–4.2) and northeastern states (aOR 2.0, 99% CI 1.1–3.6).

## Discussion

According to these findings, ≈1 in 4 TB cases reported in the United States may be attributed to recent TB transmission; this increases to 1 in 3 among US-born persons (Table 1). Our approach to identifying the proportion of reported TB attributable to recent transmission is based on the concept that epidemiologically related organisms share indistinguishable genotypes, whereas unrelated organisms differ at some genetic loci (*8*). TB cases that occur in spatial clusters and share indistinguishable genotypes are thought to be caused by recently transmitted TB infection; those with nonclustered genotypes are thought to result from progression from an infection acquired >3 years in the past. In the absence of detailed data about interpersonal contact between persons, relying on genotype and on place and time data routinely collected during surveillance activities becomes imperative to assessing recent transmission at a national level. This goal was achieved by using the established infrastructure of NTSS and TB genotyping, universally accessible to TB programs through NTGS, to capture 72% of all cases with culture-positive results over a 5-year period.

Spatial scanning provides a new insight into TB transmission that is independent of jurisdictional or geopolitical boundaries. This nationally representative study incorporated spatial concentration as a core element for defining recent TB transmission. Previous studies were limited to clustering definitions confined to a single jurisdiction (*9**–**11**,**14**,**15*), state, or province (*28**,**29*), or incomplete sampling of an entire nation (*13*,*30*). The proportion of cases representing recent TB transmission varied considerably by cluster definitions based on geopolitical borders. If a national clustering definition was used, up to 80% of culture-positive cases would be attributed to recent TB transmission. If a state-based definition or county-based definition was used, up to 57% and 39% of culture-positive cases, respectively, would be attributed to recent TB transmission. Although which definition most accurately represents recent TB transmission is unclear, a clustering definition based on geospatial concentration appears to be the most conservative and is not subject to the potential misclassification of political boundaries. The limitation of using these boundaries can be best exemplified by known inter-jurisdictional TB outbreaks that crossed geopolitical borders (*31*). Because the proportion of recent TB transmission may be a reflection of the success of control measures, accurately assessing this quantity is of considerable public health importance.

Estimating recent TB transmission also depends on the duration of the study period (*16*). Other studies have shown increasing clustering proportions as the duration of the study increases, with a plateau effect after 3 years (*12**,**13**,**17**,**32**,**33*). The annual proportion of isolates with a new strain identified in the United States during this study period did plateau (data not shown), suggesting a similar phenomenon and potential influencing factor in the long-term estimation of TB genotype clustering nationwide. Using consecutive, overlapping scanning windows that incorporate 3-year intervals maximizes the probability that spatial and temporal clustering represent localized, recent TB transmission within this large and comprehensive dataset. As NTGS continues to mature and grow over time, adjusting for temporal clustering will become essential when estimating recent TB transmission.

Consistent with other published reports from countries with a low incidence of TB, the characteristics of local birth, male sex, minority race, substance abuse, and homelessness were associated with recent TB transmission (*17**,**18**,**33*). These findings highlight the fact that TB may be harder to eliminate among populations characterized by these factors (*34*). The large proportion of cases attributable to recent TB transmission among minorities, persons who abuse substances, and those who are homeless suggests that limited access to routine health screenings, resulting in delayed diagnoses, may extend infectious periods and rates of TB transmission. Indeed, TB patients who use illicit substances and abuse alcohol have been found to be more contagious (*24*).

In low-incidence, high-resource countries, efforts to control recent TB transmission are based largely on contact investigation, yet for many reasons, contact investigations may not be sufficiently intensive or comprehensive, even in successful TB control programs (*35*). Every case of TB began when a person came into contact with a person with contagious TB. Therefore, it follows that clusters of case-patients representing recent TB transmission could be averted through improved contact investigation efforts. Contact investigations are multistep processes in which exposed contacts are systematically evaluated on the basis of the amount of time spent with an infectious person, the environmental conditions of exposure venue, and the contact's intrinsic predisposition for infection or disease (*36*). Numerous studies have demonstrated that eliciting names of contacts is neither optimally effective nor sufficient to interrupt TB transmission among high-risk groups, such as the homeless and persons who abuse substances (*1**,**24**,**37**,**38*). The potential for uninterrupted TB transmission is further exacerbated by the poor yield of name-based contact investigations among these populations. Locations are as important as named contacts when investigating recent transmission. A recent study found that 81% of case-patients involved in a multiyear TB outbreak lived in close geographic proximity (*38*). Spatial scanning methods may assist with identification of specific clusters representing ongoing transmission that could benefit from targeted location-based interventions. Using spatial scanning methods to determine locations with high concentrations of both spatial and genotype clustering may be an effective way to prioritize resources to intervene in populations with high rates of TB transmission.

This study does have limitations. First, isolate submission for TB genotyping is not universal; thus, the database, although large, did not contain all reported case-patients with culture-positive TB during the study period. Clinical, demographic, and epidemiologic characteristics of patients without TB genotyping data did not differ statistically from those with TB genotyping data (data not shown). Second, spatial and genotype clustering serves only as a proxy for recent TB transmission in the absence of detailed data on interpersonal connections between case-patients. Because of dynamic migration patterns within the United States, these methods may fail to ascertain cases that are due to recent transmission when a putative source case-patient moves or if exposure occurred outside the range of spatial scanning. Increased global migration has influenced the epidemiology of TB in the United States as well. Recent immigrants who became infected with a particular genotype elsewhere may resettle in the same neighborhood and, when TB develops after resettlement, it may falsely be considered recent TB transmission. Third, although spoligotyping and 12-locus MIRU-VNTR have good discriminatory power, these methods may not provide the resolution necessary to differentiate evolutionarily close strains (*39**,**40*). The introduction of an expanded panel of 24 MIRU-VNTR loci in 2009 to NTGS may reduce this misclassification in the future (*40*). It is also critical to note that TB transmission dynamics are multifactorial. TB genotype clustering may overestimate transmission. Consideration of patient characteristics, transmission venues, and temporality may better clarify recent transmission.

The integration of NTGS into routine public health practice and surveillance has led to the establishment of molecular surveillance of *M. tuberculosis* in the United States (*20*). With improved access to and rapid dissemination of genotyping information, it may be possible to more effectively identify some cases of TB transmission. Yet, TB genotyping, and likely future molecular advancements do not alter real-time public health action. Rather recent transmission can only be prevented by implementing thorough contact investigation and ensuring that subsequent preventive treatment is completed among those identified at highest risk of undergoing a progression from infection to TB disease. If such practices had been successfully followed, as many as one third of all reported TB cases in US-born patients may have been prevented, especially among high-risk populations, such as persons with substance abuse disorders, those experiencing homelessness, or both. Greater attention and resources are needed to develop, implement, and evaluate interventions to control and prevent transmission among these populations. As the United States continues toward TB elimination, understanding transmission dynamics among high-risk populations and establishing new strategies for rapidly detecting and effectively responding to these transmission events will enhance the progress toward achieving this target.
